# Estrogen downregulates CD73/adenosine axis hyperactivity via adaptive modulation PI3K/Akt signaling to prevent myocarditis and arrhythmias during chronic catecholamines stress

**DOI:** 10.1186/s12964-023-01052-0

**Published:** 2023-02-23

**Authors:** Marie Louise Ndzie Noah, Gabriel Komla Adzika, Richard Mprah, Adebayo Oluwafemi Adekunle, Stephane Koda, Joseph Adu-Amankwaah, Yaxin Xu, Kouminin Kanwore, Prosperl Ivette Wowui, Hong Sun

**Affiliations:** 1 Department of Physiology, Xuzhou Medical University, 209 Tongshan Road, XuzhouJiangsu, 221004 China; 2 Jiangsu Key Laboratory of Immunity and Metabolism, Department of Pathogenic Biology and Immunology, Xuzhou Laboratory of Infection and Immunity, Xuzhou Medical University, Xuzhou, China; 3 Public Experimental Research Center, Department of Neurobiology and Anatomy, Xuzhou Medical University, Xuzhou, China

**Keywords:** Catecholamine stress, Cardiac inflammation, CD73, Adenosine, Estrogen

## Abstract

**Background:**

During myocardial damage, the sex hormone estrogen and CD73, the main enzyme that converts AMP into adenosine, are cardioprotective molecules. However, it is unclear how these two molecules work together to provide cardioprotection. The current study aimed to elucidate the interaction between estrogen and CD73 under chronic stress.

**Methods:**

Ovariectomy and SHAM operations were done on FVB wild-type (WT) female mice. Two weeks after the operation, the mice were treated with daily isoproterenol (10 mg/kg/day) injections for 14 days. The effect of E2 on relevant cardiac injury biomarkers (BNP, ANP), myocardial morphology (cardiomyocyte surface area), electrocardiography, CD73 protein expression and activity, and macrophage (CD86 + and CD206 +) infiltrations were assessed. In vitro, H9C2 cells were treated with 1 nM of estrogen and 10 mM APCP (CD73 inhibitor α, β-methylene adenosine-5'-diphosphate), 10 µM isoproterenol and 20 µm LY294002 (PI3K inhibitor) for 24 h and western blot was done to elucidate the mechanism behind the effect of estrogen on the CD73/adenosine axis.

**Results:**

Estrogen deficiency during chronic catecholamine stress caused myocardial injury, thereby triggering the hyperactivity of the CD73/adenosine axis, which aggravated myocarditis, adverse remodeling, and arrhythmias. However, estrogen normalizes CD73/Adenosine axis via the upregulation of PI3K/Akt pathways to prevent adverse outcomes during stress. In vivo results showed that the inhibition of PI3K significantly decreased PI3K/Akt pathways while upregulating the CD73/adenosine axis and apoptosis.

**Conclusion:**

Estrogen’s pleiotropy cardioprotection mechanism during stress includes its normalization of the CD73/Adenosine axis via the PI3K/Akt pathway.

**Video Abstract**

**Supplementary Information:**

The online version contains supplementary material available at 10.1186/s12964-023-01052-0.

## Background

Catecholamines are neurotransmitters linked to cardiovascular diseases (CVDs) [1]. Under normal circumstances, these hormones stimulate the β-adrenergic receptors (β-ARs) as part of the fight-or-flight response [2]. However, under stress conditions, βARs functions are dysregulated and cause several damages such as cardiac hypertrophy and apoptosis [3, 4]. Also, Cardiomyocyte apoptosis is observed to be elevated in severe hypertrophic conditions leading to a reduction in cardiac contraction and function [5–7]. The incidence of CVDs has also been associated with variations in estrogen levels (E2) among menopausal women. Recent data reveal that E2 has a crucial role in regulating several processes (immunomodulation, anti-apoptosis) in the heart via its non-genomic and genomic signaling [8, 9]. The cardioprotective effect of E2 has been demonstrated in premenopausal women having a lower risk of CVDs [8]. The possible underlying mechanism of this beneficial effect has been well elucidated. GPR30 signals increased cardiomyocyte inotropy (rapid response) during physiological conditions via the Gs/cAMP/PKA/EPAC pathway. In contrast, during chronic catecholamine stress (CCS), this signaling shifted to the Gi/PI3K/Akt pathway to repress inotropy and apoptosis [10]. Besides GPR30, estrogen receptors α (ERα) and β (Erβ) exert a non-rapid estrogenic response via the activation of transcriptional factors [11].

CD73 is a glycosylphosphatidylinositol (GPI)-linked membrane-bound glycoprotein and has been reported to be a cardioprotective protein [12, 13]. This protein is found on various cells (myocytes and immune cells) and is critical for maintaining cardiomyocyte tissue integrity and immune homeostasis. CD73 interacts with CD39, an upstream signaling molecule, to produce adenosine from extracellular nucleotides (ATP and ADP) [14]. By interacting with purinergic receptors (A1, A2A, A2B, and A3AR) associated with the G protein receptors, adenosine has been demonstrated to reduce the cardiac proinflammatory response [15]. Nevertheless, the connection between E2 and extracellular purinergic metabolism is poorly understood. Recent studies have shown that E2 inhibits the CD73/adenosine axis, which may have protective effects in breast cancer and vascular diseases [16, 17]. However, the direct effects of E2 on the CD73/ adenosine axis during myocardial injury are not well elucidated. Hence, this study aimed to examine the interaction between E2 and CD73 and the underlying mechanism involved in their cardioprotective function during chronic stress.

To check our hypothesis, we explored the effects of E2 deficiency and its replacement on the extracellular purinergic metabolism (CD73/Adenosine) during CCS in FVB wild-type (WT) female mice and the H9C2 cell line. We further elucidate the mechanism by which Estrogen regulates CD73 expression and activity.

## Materials and methods

### Experimental animals and models

FVB wild-type female mice were used (n = 6 to 8 mice/group)**,** and all required animal experimental rules were strictly observed. As detailed previously, an ovariectomy (OVX) or SHAM operation was performed in 2-month-old females [18]. Briefly, Mice were anesthetized with 100 mg/kg of esketamine (esketamine hydrochloride; H20193336), and ovaries were exposed through an abdominal approach and either resected after clipping the blood vessels or left in place (sham operation). The muscle and skin of the stomach were sewed. After two weeks post-surgery, it was observed that complete wound healing was evidenced with the detachment of the suture. OVX mice were then divided into three specific groups (1) OVX + ISO group: this group received a subcutaneous (s.c) injection of 10 mg/kg/day isoproterenol (ISO) (160504; Sigma) for 14 days to cause stress-induced myocardial remodeling. Also, the same treatment was administered to the Sham surgical mice (SHAM + ISO);(2) OVX + E2 group: this group received an s.c injection of 40 µg/kg/day E2 (E2758; Sigma) for 14 days as previously demonstrated[19, 20] (3) OVX + ISO + E2 group: This group received a combination treatment of ISO and E2 for 14 days. At the end of in vivo experiment, we had six distinct groups; (1) SHAM Group, (2) OVX Group, (3) OVX + E2 Group, (4) SHAM + ISO Group, (5) OVX + ISO Group, (6) OVX + ISO + E2 Group.

### Electrocardiography

At the end of all animal models, electrocardiography (ECG) data acquisitions were carried out by utilizing PowerLab systems’ 3-lead monopolar needle electrode (ADInstruments), as described previously [21].

### Cells culture and treatment

H9C2 was obtained from the National Collection of Authenticated Cell Culture, China. The cells were cultured in a DMEM medium supplemented with (v/v) 10% heat-inactivated FBS and 1% penicillin for 2 to 3 days until they reached (70–80) % at 37 °C in a humidified atmosphere of 5% CO_2_. The following cells were then divided into 7 groups and sub-cultured for another 2 to 3 days. On day 2 or 3, the cells received the following treatment for 24 h. (1) Control groups, (2) ISO: Treated with 10 µM of ISO, (3) APCP groups: These groups received CD73 inhibitor α,β-methylene adenosine-5'-diphosphate (10 mM), (4) E2 groups: Treated with 1 nM of E2, (5) Combination treatment groups E2 + ISO: Cells were first incubated with 1 nM of E2 for 30 min, then ISO was added, (6) E2 + APCP group: These groups received an E2 pre-treatment for 30 min before continuing the remaining treatment, (7) E2 + ISO + LY294002: 30 min of pre-treated with E2, another 30 min were added for LY294002 (20 µM) incubation before proceeding with ISO treatment. For immunocytochemistry assay, the cells were seeded in plates of 24 wells containing glass coverslips (final volume of 2 ml/well) and were allowed to grow for 3-4 days to reach the desired confluence (> 80–90%). The experiments were conducted on day 3 or 4 after the cells received the aforementioned treatments for 24 h. The supernatants from all groups were collected and cryopreserved immediately at -80℃ for further experiments.

### Western blotting analysis

Total protein was extracted from the heart and H9C2 cells using a homogenizer, then treated with reducing agents, denatured at 100℃ for 10 min, and separated by gel electrophoresis as previously described[10]. The transferred protein bands were blocked with 1% bovine serum albumin and incubated in the following primary antibodies listed in Table 1 at 4 °C overnight. Immunoblot visualizations were performed using enhanced chemiluminescence (Tanon, Shanghai, China). The relative expressions of the GAPDH were used to measure and assess the protein bands.

**Table 1:** Antibody used in the study

No	Antibody	Company	Catalog No
1	CD73	Abcam	Ab175396
2	Akt (pan) (11E7) Rabbit mAb	Cell Signalling Technology	4685S
3	Phospho-Akt (Ser473) (D9E) XP® Rabbit	Cell Signalling Technology	4060S
4	GAPDH	Proteintech	10,494–1-AP
5	PI3 Kinase p110α Antibody	Cell Signalling Technology	4255S
6	Goat-anti-Rabbit Secondary Antibody	Proteintech	SA00001-2
7	Phospho-PI3KP85α/γ/β-Y467/Y199/Y464Rabbit pAb	ABclonal	AP0854
8	Cleaved Caspase-3 (Asp175) (D3E9) Rabbit mAb	Cell Signalling Technology	#9579
9	phosphatidylinositol 3 (PI3) kinase inhibitor	Sigma	LY294002

### Enzyme-linked immunosorbent assay

Myocardial lysate from in vivo models (n = 6/group) was used to assess the concentrations of proinflammatory cytokines IL-6(Proteintech; KE10007) and TNF-α (Proteintech; KE10002), the anti-inflammatory cytokine IL-10(KE10008; Proteintech) and heart injury biomarkers BNP (JL12884; Jianglai biology, Shanghai), and ANP (JL20612; Jianglai biology, Shanghai). ELISA was performed in triplicates as per the instructions of the manufacturer.

### Adenosine assay

Lysate from in vivo models (n = 4/group) and cell culture media supernatants from treated H9C2 cells were used to assess the adenosine concentration by fluorometric assay (MET-5090, Cell BioLabs) following the manufacturer’s instructions. Adenosine activity content was measured at 570 nm using a microplate reader.

### Histology, immunohistochemistry, and immunofluorescence staining

To assess the histopathological changes in heart mice, the frozen heart tissue was serially sectioned at 4 μm for wheat germ agglutinin (WGA) (W11261; Thermo Fisher Scientific), and the staining was performed following the manufacturer’s instructions.

For IHC staining, sections of frozen myocardia were used for the staining of CD86 (Bioss, Woburn, MA, United States; BS-1035R) and CD206 (Abcam; ab8918) to assess the extent of cardiac macrophages infiltration. For that, the heart tissue was hydrated, and non-specific antibody binds were prevented by flooding the slides with H_2_O_2_ for 10 min and then stopping with 5% BSA for 30 min. The slides were then incubated overnight with primary anti-CD86 and anti-206. The following day, tissues were incubated with biotinylated goat anti-rabbit IgG and Streptavidin peroxidase for 25 min each, followed by DAB staining and hematoxylin counterstaining. The slides were dried in xylene and mounted for microscopy (IX 71, Olympus, Tokyo, Japan).

For IF, Cultured H9C2 cells were fixed and permeabilized with pre-chilled methanol–acetone (ratio 1:1). Non-specific antibody binds were stopped with 1% BSA in PBS for 1 h. H_9_C_2_ were then incubated with CD73 primary antibody (ab175396; Abcam) and cleaved caspase 3( CC3) primary antibody (#9579; Cell Signaling Technology) at 4^0^C overnight, washed with PBS and probed with R-PE-conjugated secondary antibody (SA00008-2; Proteintech) at room temperature for 1 h. Cells were washed with PBS and incubated with DAPI nuclei staining for 3 min, followed by imaging and an assessment of the ratios CD73 and CC3 expressions in the nucleic and cytoplasm. Imaging of all tissue stained was analyzed using ImageJ (1.53a version; National Institute of Health, Bethesda, MD, United States).

### Statistics and reproducibility

Sample sizes were determined by Power analysis, and statistical assessment was performed with GraphPad Prism 5.0 (GraphPad Software, San Diego, CA, United States). All data are expressed as mean ± SEM. Comparing three or more groups was done with one-way ANOVA, while two-way ANOVA was used to analyze grouped data. Post hoc analyses using Tukey’s multiple comparisons test *p* < 0.05 were deemed statistically significant. Gaussian distribution was ensured, and homogeneity of variance were tested (Levene’s median test—all *p*-value > 0.05) as well as sphericity controlled (Bartlett's test—all *p*-value > 0.05). All experiments are represented by multiple biological replicates.

## Results

### Estrogen mitigates cardiac morphological alteration and arrhythmias during CCS

**Fig. 1:**
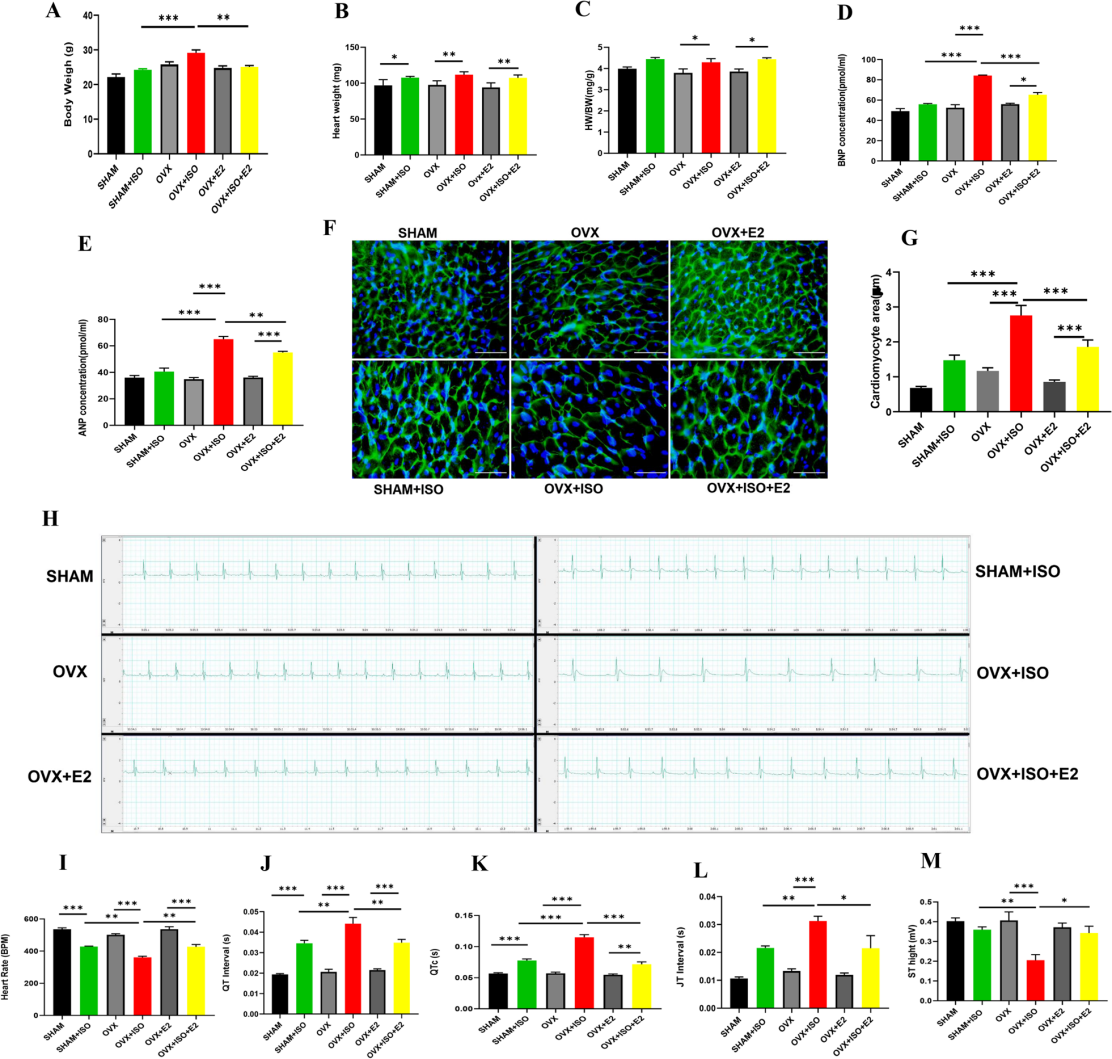
Morphometric and myocardial injury indexes change After 14 days of CCS. **A**–**C** Graphical presentations of morphometric data demonstrate alterations in ratio of body weight/heart weight (HW/BW), body weight (BW), and heart weight HW (n = 5–6). **D**, **E** Effect of E2 on plasma BNP and ANP concentration (n = 5–6). **F**, **G** Representative microscopic images of wheat germ agglutinin (WGA) and graphical illustration of measured cardiomyocyte area. **H**–**M** Representative electrocardiography (ECG) and graphical presentation of ECG parameters including; Heart Rate, QT, corrected QT (QTc), JT Interval, and ST Height (n = 5 mice per group). One-way ANOVA, followed by Turkey’s multiple comparison test. ***p < 0.001; **p < 0.01; *p < 0.05).

We observed that E2 deficiency facilitated body weight (BW) increment, which got aggravated during stress (OVX + ISO) (Fig. 1A). The morphometric results demonstrate that the injection of ISO slightly increased the heart weight (HW) and, consequently, HW/BW ratio in SHAM + ISO compared to the SHAM (control group) (Fig. 1B, C). Also, the results showed that CCS significantly increased the ratio HW/BW in the OVX + ISO group, which showed statistical significance when compared to OVX. However, the presence of exogenous estrogen (E2_Exo_) in the OVX + E2 significantly decreased the ratio of HW/BW compared to OVX + ISO + E2**.** Assessing BNP and ANP levels showed slight increases in SHAM + ISO compared to the SHAM group (Fig. 1D, E). Nonetheless, the deficiency of estrogen in the OVX + ISO group caused significant increases in both BNP and ANP expressions. Even so, the supplementation of E2_Exo_ (in the OVX + ISO + E2 group) significantly decreased the expressions of these natriuretic peptides**.** Furthermore, by using WGA staining to examine the cardiomyocyte surface area, we observed that the OVX + ISO hearts had excessive myocyte hypertrophy compared to the OVX + ISO + E2 and SHAM + ISO hearts (Fig. 1F, G). Next, we sought to ascertain the state of cardiac electrical activities to determine incidents of arrhythmias. The ECG data showed that CCS induces decreases in heart rates (HR); however, unlike in SHAM + ISO and OVX + ISO + E2, the estrogen deficiency in OVX + ISO permitted further depression in HR. Similarly, we observed substantial prolongation of QT, corrected QT, and JT interval, as well as ST height depression in OVX + ISO mice, compared to SHAM + ISO and OVX + ISO + E2 mice (Fig. 1H, M). The significant alterations in these indexes are indicative of arrhythmias in the OVX + ISO mice; even so, less severities were observed in SHAM + ISO and OVX + ISO + E2 mice.

### Estrogen downregulates CD73/adenosine axis during CCS

**Fig. 2:**
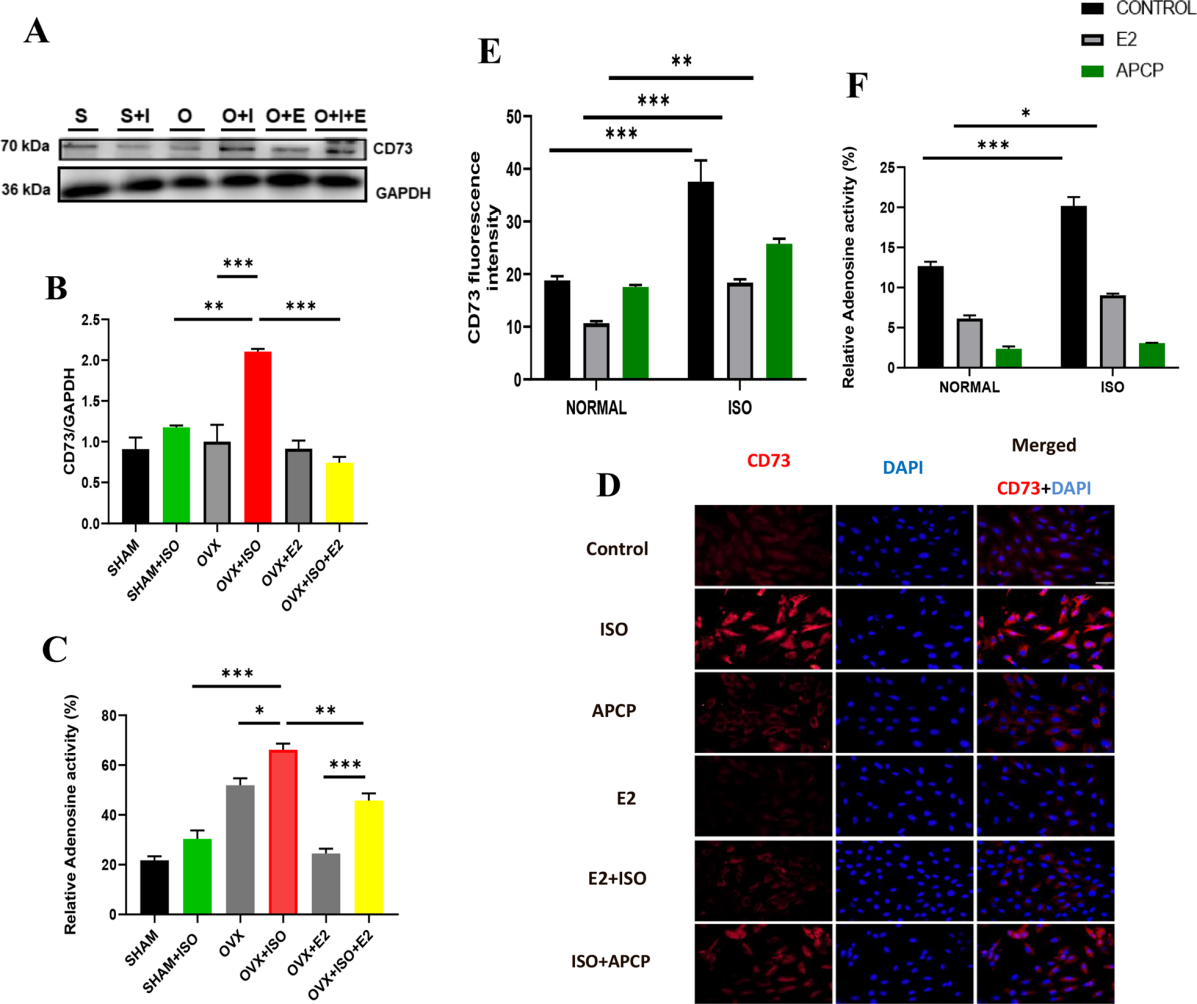
Effect of E2 on CD73/adenosine axis: **A**–**C** Representative immunoblots, graphical presentations of CD73 expression and ELISA analysis of Extracellular Adenosine level (n = 6–7 mice/group). S = Sham; S + I = Sham + ISO; O = OVX; O + I = OVX + ISO; O + E, OVX + E2; O + E + I = OVX + ISO + E2. **D** Representative immunofluorescence of CD73expression, nuclei (DAPI) **E** The plotted average values of CD73 expression from each group (n = 100–150 cells per dish per 4–6 repeats per treatment group). Color channels were adjusted in the merged images to enhance the visualization of all the respective fluorescence dyes. **F** Analysis Adenosine activity. Data were analyzed using One –way and Two-way ANOVA, followed by Tukey’s post hoc analysis. ***p < 0.001; **p < 0.01; *p < 0.05

To affirm the role of E2 on the CD73/ adenosine axis during CCS. CD73 expression and activity were assessed in OVX animals treated either with a single dose of E2, ISO, or a combination of both treatments. The results showed that estrogen deficiency (in OVX + ISO mice) during CCS significantly increased CD73 expression and adenosine activity compared to the OVX group. Furthermore, compared to the OVX + ISO group, the presence of both endogenous (E2_Endo_) and exogenous E2 under stress significantly reduces adenosine activity and CD73 expression in SHAM + ISO and OVX + ISO + E2, respectively. In the absence of stress, both endogenous (in SHAM) and exogenous (in OVX + E2) E2 did not affect CD73/adenosine expression (Fig. 2A–C). To better understand the impact of E2 on the CD73/adenosine axis, H9C2 cell line was treated with ISO, CD73 inhibitor (APCP), and estrogen for 24 h. Our results indicated that in the absence of stress, the expression of CD73 slightly decreased in E2 compared to APCP and control groups.

On the other hand, the presence of stress increases the expression of CD73 in the APCP + ISO group, but those effects were more obvious in the ISO groups compared to the E2 + ISO group (Fig. 2D, E). Next, the activity of adenosine was lower in APCP and E2 groups compared to the control group. Furthermore, ISO treatment significantly increases adenosine activity (in the ISO group) compared to E2 + ISO and APCP + ISO groups (Fig. 2F). This suggests that E2 treatment was able to decrease both the activity and expression of CD73.

### Estrogen suppresses maladaptive myocardial inflammatory responses during CCS

**Fig. 3:**
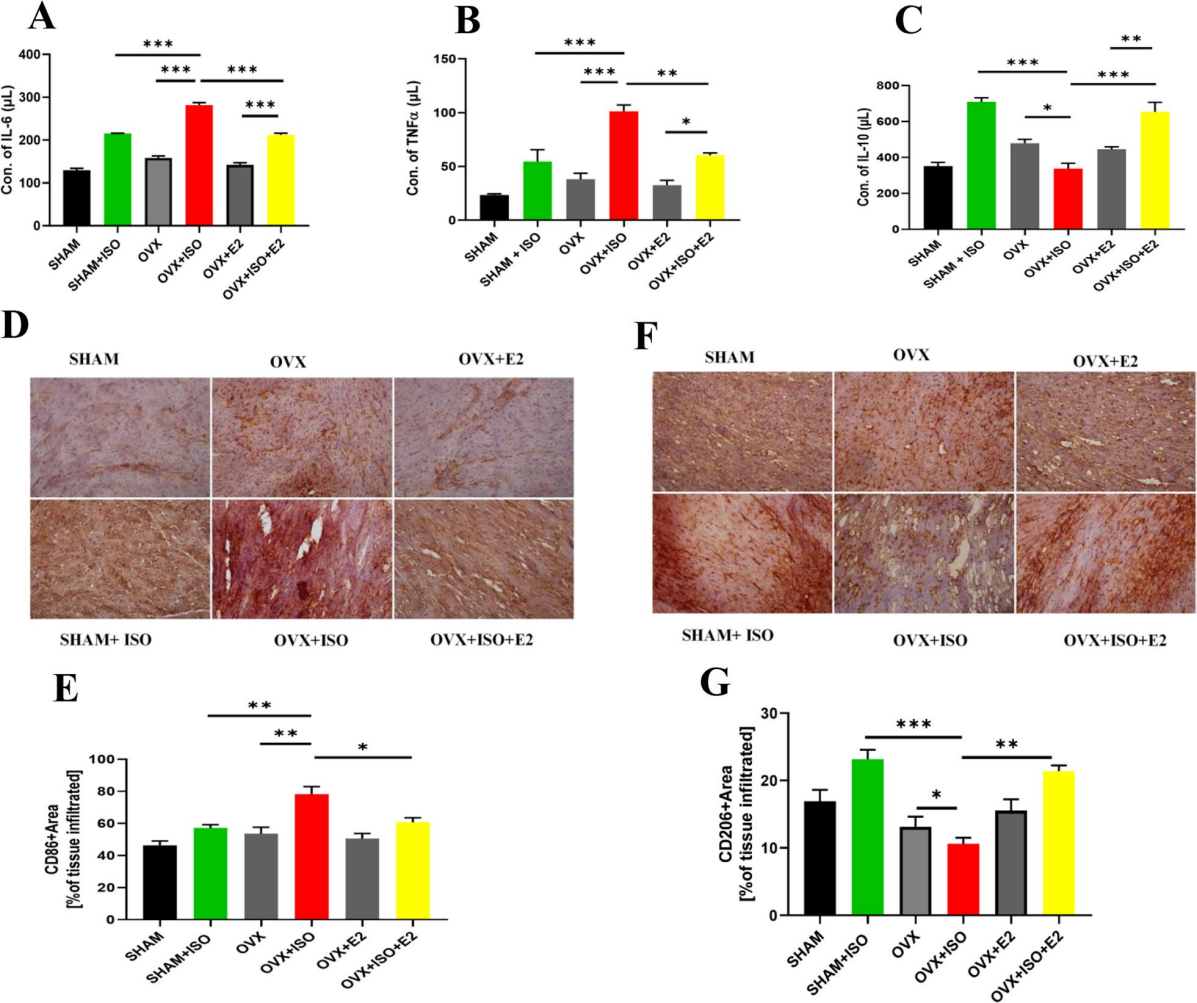
Analysis the effect of E2 on myocardial inflammatory responses during CCS. **A**–**C** Expressions of inflammatory markers IL-6, TNFα, and IL-10 were assessed by ELISA (n = 4–6 hearts per group). **D**–**G** Representative immunohistochemical staining and graphical presentation of macrophage CD86 and CD206-positive cells assessed from the myocardial sections (n = 4 hearts per group). Data are presented as mean ± SEM; ***p < 0.001; **p < 0.01; *p < 0.05

Chronic stress has been demonstrated to increase myocardial inflammation, a mediator in the progression of heart failure [22]. Hence, we assessed the effect of E2 on myocardial inflammatory response during CCS. Enzyme-Linked Immunosorbent Assay showed that ISO administration (in SHAM + ISO) slightly increased IL-6, TNF-α, and IL-10 secretions compared to the SHAM group. The effect of ISO was more evident in OVX + ISO group, with IL-6 and TNF-α secretions significantly upregulated, while IL-10 was downregulated compared to the OVX group. Overall, both endogenous and exogenous estrogens significantly reduced IL-6 and TNF-α secretions and maintained the regulation of IL-10 in SHAM + ISO and OVX + ISO + E2 compared to the OVX + ISO group (Fig. 3A–C). Subsequent macrophages CD86 + and CD206 + IHC staining demonstrated that injection of ISO slightly elevated the number of CD86-positive (proinflammatory) macrophages infiltrating the myocardia in SHAM + ISO compared to SHAM. Although the absence of estrogen during stress caused massive infiltration of macrophages CD86-positive cells, CD206-positive (anti-inflammatory) cell infiltrations are significantly reduced in the OVX + ISO group compared to OVX. In contrast, the supplementation of E2_Exo_ (in OVX + ISO + E2 mice) significantly decreased the number of CD86 macrophages infiltrating the myocardia while keeping CD206 upregulated, similar to SHAM + ISO (which has E2_Endo_ present) (Fig. 3D–G).

### Estrogen adaptively modulates CD73/adenosine axis via PI3K/Akt pathway

**Fig. 4:**
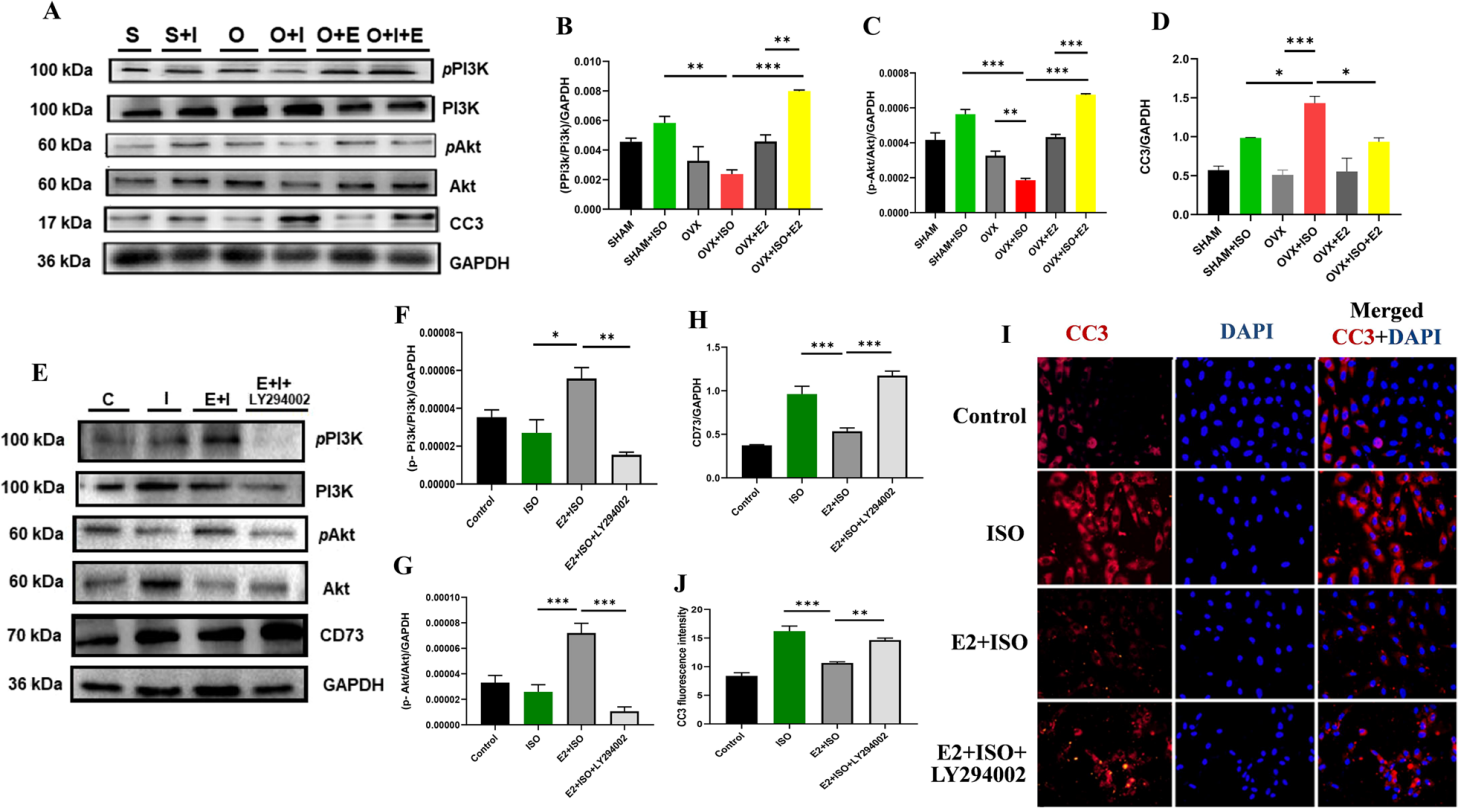
In vivo results of estrogenic adaptive regulation of CD73/adenosine axis via PI3K/Akt pathways during CCS. **A**–**D** Representative immunoblots and graphical presentations of cleaved caspase 3, PI3K, p-PI3K and Akt, p-Akt, were assessed from Mice. S = Sham; S + I = Sham + ISO; O = OVX; O + I = OVX + ISO; O + E, OVX + E2; O + E + I = OVX + ISO + E2. **E**–**H** In vitro results of inhibition of PI3Kby LY294002. Representative immunoblots and graphical presentations of, PI3K, p-PI3K, Akt, p-Akt and CD73. **I**, **J** Representative immunofluorescence and plotted average values of CC3 expression. Data are presented as mean ± SEM; ***p < 0.001; **p < 0.01; *p < 0.05

According to previous studies PI3K/Akt signaling pathway is involved in the regulation of apoptosis [23]. Apoptotic cells release extracellular ATP through pannexin 1, which is later converted to adenosine by ectonucleotidases (CD39, CD73) [24]. To study the pathway by which E2 regulated the CD73/adenosine axis during CCS. We checked the effects of estrogen and ISO on phosphorylated PI3K (p-PI3K), Akt (p-Akt), and caspase 3(CC3) during CCS. We found that E2 deficiency decreased the expression of phosphorylated PI3K (p-PI3K) and Akt (p-Akt) in the OVX + ISO group, whereas CC3 protein expression levels significantly increased compared to the OVX group. However, E2_Exo_ (in OVX + ISO + E2 mice) and E2_Endo_ (in SHAM + ISO) reversed the prior mentioned phenomena. PI3K and Akt phosphorylation increased significantly, but CC3 expression decreased dramatically (Fig. 4A–D).

To clarify the mechanism by which E2 controls CD73/adenosine axis via PI3K/Akt signaling during CCS. We tested whether E2 uses PI3K to modulate the CD73/adenosine axis during CCS using LY294002(PI3K inhibitor). The suppression of PI3K significantly decreased p-PI3K and p-Akt while upregulating the CD73/adenosine axis and CC3 in the E2 + ISO + LY294002 group compared to the ISO + E2. In addition, the presence of E2 (in the E2 + ISO group) significantly increases p-PI3K and p-Akt while downregulating the CD73/adenosine axis and CC3 compared to the stress group (ISO) (Fig. 4E–I). The findings suggest estrogen downregulates the CD73/adenosine axis through PI3K/Akt.

## Discussion

This study describes the direct impact of E2 on the CD73/adenosine axis during CCS and the mechanisms behind this relationship. We found that CD73/adenosine axis and proinflammatory cytokines decrease significantly in the presence of E2. Interestingly, decreased CD73/adenosine axis expressions were associated with increased p-PI3K and p-Akt levels and decreased CC3 expression. Also, in vitro, we found that PI3K/Akt signaling is required for E2 downregulation of the CD73/adenosine axis.

Chronic catecholamine stress is a risk factor for CVDs, and premenopausal women are less susceptible to developing than men [25]. This relative immunity has been attributed to the cardioprotective role of estrogen [8]. However, a decline in estrogen levels during menopause can lead to a detrimental effect on the heart. In this study, the morphometric data shows that the absence of E2 under stress (in OVX + ISO) results in cardiomyocyte hypertrophy which was established by an increase in BW, HW, HW/BW ratio, and cardiomyocyte surface area. In line with our results, E2 deficiency was reported to cause disorders of lipid metabolism, which inadvertently contributes to an increase in the heart's size and weight gain due to increased lipid accumulation in the body [26, 27]. Also, the increased HW, HW/BW ratio when E2 is low was a cause of enhanced epicardial adipose and cardiomyocyte hypertrophy during stress [28]. Surprisingly, it has been demonstrated that an increase in epicardial fat directly contributes to the proinflammatory responses by harboring inflammatory cells and impeding M1 macrophages' polarization into M2 macrophages (anti-inflammatory and reparative phenotype), thereby aggravating myocarditis and maladaptive remodeling [29].

Further evaluation of BNP and ANP levels also supports the existence of cardiomyocyte hypertrophy (in OVX + ISO), which is shown by the upward regulation of these two natriuretic peptides. Consistent with these findings, ANP and BNP have been well demonstrated to be vasodilation natriuresis, and elevated plasma levels of both natriuretic peptides are linked to myocardial damage resulting in cardiomyocyte hypertrophy [30–33]. Nevertheless, E2 or its supplementation prevented the morphological alteration, as demonstrated here [34]. These aforementioned morphological changes tend to induce arrhythmia – as such, ECG were performed to ascertain the state of cardiac electrical activities. Consistently with Hou et al.’s findings [35], estrogen deficiency during CCS (in OVX + ISO) encouraged further decreases in HR and aggravated arrhythmia as demonstrated by the significant prolongation of QT, corrected QT, and JT interval, as well as ST height depression. Remarkably, the severity of these phenomena were mitigated in SHAM + ISO and OVX + ISO + E2 mice, which had E2_Endo_ and E2_Exo_, respectively, presented. Taken together, these findings consolidated the evidence that estrogen mitigates cardiac morphological alteration and severe arrhythmias during stressful conditions.

Following myocardial injury, immune cells infiltrate into the failing heart. This phenomenon is exacerbated when E2 is deficient and has been attributed to the fact that E2 adaptive immunoregulation in the myocardia [36]. Like E2, CD73 has also been implicated in regulating myocardial inflammation during stress [13]. However, the interaction between this cardioprotective molecule during myocardial injury is unclear. Our results showed that E2 deficiency during stress permitted maladaptive cascades in the CD73/adenosine axis, which resulted in cardiomyocyte necrosis indicated by elevated levels of proinflammatory cytokines (IL-6 and TNFα) and massive numbers of CD86 + infiltration. Consistently, prior research has shown that CD73 produces adenosine acutely during injury as a homeostatic response to the increased cardiac inflammation; however, its excessive production can promote tissue damage [37, 38]. Furthermore, in correlation with the extent of cardiomyocyte necrosis, studies have reported that cardiomyocyte necrosis following injury aggravates proinflammatory responses [39, 40]. In contrast to E2 deficiency, E2_Endo_ presence (in SHAM + ISO) and E2_Exo_ supplementation (in OVX + E2 + ISO) significantly decreased CD73/adenosine hyperactivity. This prevented aggravated cardiomyocyte necrosis by increasing the anti-inflammatory responses (IL-10 and massive infiltration of macrophage CD206) to ensure reparative functions. Contrary to our findings, Lee J et al. have reported that only supplementation of E2 does not affect the expression of CD73, while the co-administration of E2 and progesterone decreased its expression [41]. Even so, this study was conducted in the estrous cycle in mouse uterus and not during CCS or in isoproterenol-induced cardiac injury models. Also, the duration and the dosage of E2 they applied were lessened than what we used in this study [41].

Nonetheless, our findings regarding the decreases in CD73/adenosine axis by E2 conformed with previous studies [16, 42]. In addition, this finding also supports the evidence that estrogen signaling facilitates adaptive immune response by increasing the synthesis of anti-inflammatory compounds to resolve inflammation [22, 43]. To complement our finding, in vitro investigations of H9C2 cells treated with APCP and/or E2 during stress showed that while APCP only inhibited adenosine production, E2 modulated their activity and expressions close to their physiological levels to facilitate their adaptive functions. Hence, it is speculated that E2 adaptatively regulates CD73/adenosine axis during stress, which might have facilitated the downregulation of maladaptive proinflammatory in SHAM + ISO and OVX + E2 + ISO mice.

Lastly, we sought to investigate the mechanism by which E2 regulates CD73/adenosine axis during CCS. Previous studies have shown that βAR functions are deregulated under stress and cause several damages (cardiomyocyte necrosis or apoptosis), thus increasing extracellular purinergic metabolism (CD73activity) [3, 4, 44]. Similarly, E2 deficiency is associated with increased apoptosis, proinflammatory response, and impaired cardiac function due to decreased signaling via Gαi/PI3K/Akt [4, 8]. We observed that, compared to SHAM + ISO and OVX + E2 + ISO, the protein expressions of the phosphorylated PI3K/Akt were downregulated in the OVX + ISO hearts and accompanied by a significant increase in apoptosis (CC3 expression). Also, it was observed that an increase in cardiomyocyte apoptosis was correlated with overexpression CD73/adenosine axis. Meanwhile, during stress, E2 presence increased PI3K/Akt phosphorylation, as previously demonstrated here [8, 45], while sustaining and normalizing the expression and the activity of the CD73. Hence, these findings addressed our initial speculation of the adaptive impact of E2 on CD73/Adenosine and further provided evidence that the underlying signaling was mediated by PI3K/Akt. Similar to the in vitro experiment, H9C2 cells treated with LY294002 and E2 treatments during stress demonstrated increased PI3K/Akt phosphorylation in the E2 + ISO group while downregulating CD73/adenosine and apoptosis. However, when PI3K was inhibited, we observed increased apoptosis and CD73/adenosine axis hyperactivity. Concordantly, it has been shown that increases in apoptosis lead to upregulations in the release of extracellular ATP and adenosine, thus resulting in increased inflammatory responses [15]. This finding indicates that E2 elicits a cardioprotective effect via downregulating the CD73/adenosine axis. Therefore, from both in vivo and in vitro investigations, we validated that E2 normalized CD73/Adenosine adaptively via PI3K/Akt signaling to circumvent adverse outcomes during stress.

## Conclusions

Altogether, this study work demonstrates that E2 deficiency during CCS aggravates myocarditis and arrhythmias. Herein, it is demonstrated that myocardial injury during stress triggers overexpression of CD73, which increases inflammatory responses and apoptosis. The findings also show that the estrogen’s pleiotropy cardioprotection mechanism during stress includes normalizing the CD73/Adenosine axis via the PI3K/Akt pathway. Owing to the clinical relevance of our finding, it is important to highlight its limitations and translational potential. While the involvement of E2 in the adaptive regulation of the CD73/adenosine axis, we did not identify which estrogen receptors were mediating the cascades. Even so, prior studies have demonstrated that activation of ERα and GPER1 receptors modulate both protein expressions and activity of CD73, while ERβ activations are involved in only CD73 activity regulations [45–47]. Also, from a clinical translational standpoint, our observations reemphasize the importance of estrogen replacement treatment during menopause since it confers cardioprotection against stressful events, injuries, myocarditis, and arrhythmias. Even so, Rossouw et al. and Michalson et al., among others, have demonstrated that it is vital to start estrogen replacement treatments within 5–6 years after menopause to fully recoup the therapeutic benefits with mitigated adverse outcomes [48, 49].

Additionally, we observed that while E2_Exo_ (in OVX + E2 + ISO) conferred cardioprotection during CCS, E2_Endo_ (SHAM + ISO) showed modestly more potency. This could be attributed to the fact that while estrogen concentrations altered according to the estrous cycling in SHAM + ISO mice, the supplemented E2_Exo_ concentration remained constant throughout the modeling, thereby might account for the moderate variations in E2_Exo_’s potency. As such, it is suggested that estrogen treatment dosages should mimic the concentrations of the estrous cycle to attain its full therapeutic effect.

## Data Availability

The datasets generated in this study have been included in this article as figures.

## Abbreviations

E2: Estrogen
ISO: Isoproterenol
APCP: CD73 inhibitor α, β-methylene adenosine-5'-diphosphate
p-PI3K: Phosphorylated PI3K
p-Akt: Phosphorylated Akt
CVDs: Cardiovascular diseases
CC3: Caspase 3
E2_Endo_: Endogenous estrogen
CCS: Chronic catecholamines stress
E2_Exo_: Exogenous estrogen
OVX: Ovariectomy
β-ARs: β-Adrenergic receptors
